# Skin Microbiome, Metabolome and Skin Phenome, from the Perspectives of Skin as an Ecosystem

**DOI:** 10.1007/s43657-022-00073-y

**Published:** 2022-10-10

**Authors:** Huizhen Chen, Qi Zhao, Qian Zhong, Cheng Duan, Jean Krutmann, Jiucun Wang, Jingjing Xia

**Affiliations:** 1 Human Phenome Institute, School of Life Sciences, Fudan University, Shanghai, 200438 China; 2 Department of Epidemiology, School of Public Health, Cheeloo College of Medicine, Shandong University, Jinan, 250012 China; 3 IUF-Leibniz Research Institute for Environmental Medicine, Düsseldorf, D-40225 Germany; 4 Greater Bay Area Institute of Precision Medicine (Guangzhou), School of Life Sciences, Fudan University, Guangzhou, 511458 China; 5 Research Unit of Dissecting the Population Genetics and Developing New Technologies for Treatment and Prevention of Skin Phenotypes and Dermatological Diseases (2019RU058), Chinese Academy of Medical Sciences, Shanghai, 200438 China

**Keywords:** Skin microbiome, Metabolome, Phenome, Microbe–microbe interactions, Ecological niches

## Abstract

Skin is a complex ecosystem colonized by millions of microorganisms, including bacteria, fungi, and viruses. Skin microbiota is believed to exert critical functions in maintaining host skin health. Profiling the structure of skin microbial community is the first step to overview the ecosystem. However, the community composition is highly individualized and extremely complex. To explore the fundamental factors driving the complexity of the ecosystem, namely the selection pressures, we review the present studies on skin microbiome from the perspectives of ecology. This review summarizes the following: (1) the composition of substances/nutrients in the cutaneous ecological environment that are derived from the host and the environment, highlighting their proposed function on skin microbiota; (2) the features of dominant skin commensals to occupy ecological niches, through self-adaptation and microbe–microbe interactions; (3) how skin microbes, by their structures or bioactive molecules, reshape host skin phenotypes, including skin immunity, maintenance of skin physiology such as pH and hydration, ultraviolet (UV) protection, odor production, and wound healing. This review aims to re-examine the host–microbe interactions from the ecological perspectives and hopefully to give new inspiration to this field.

## Introduction

The skin is considered a barrier organ against the entry of foreign physical, chemical, and biological insults, thereby maintaining the internal homeostasis of the human body. In the past decades, Human Microbiome Project (HMP) has expanded our perception of the skin as not only a piece of placid “soil” but a vast “ecosystem” that harbors a myriad of microbial inhabitants (Human Microbiome Project Consortium 2012). It has been believed that the colonization of diverse microbes resulted from millions of years of mutual adaptation and functional integration (Lousada et al. 2021), and thus the human body forms a complex, synergistic entity, termed a holobiont or meta-organism (Bosch and McFall-Ngai 2011; Rosenberg et al. 2007). The environmental and nutrient conditions define the unique microhabitats for skin microbes (Flowers and Grice 2020), and in turn, these microbes can influence their survival environment (host skin) by stabilizing, mutually beneficial host–microbe interactions (Postler and Ghosh 2017). In various disease conditions, the host–microbe interactions became imbalanced, termed “dysbiosis”, presenting various shifts in microbiome from “healthy” to “diseased” states (Thomas and Jobin 2020).

**Fig. 1:**
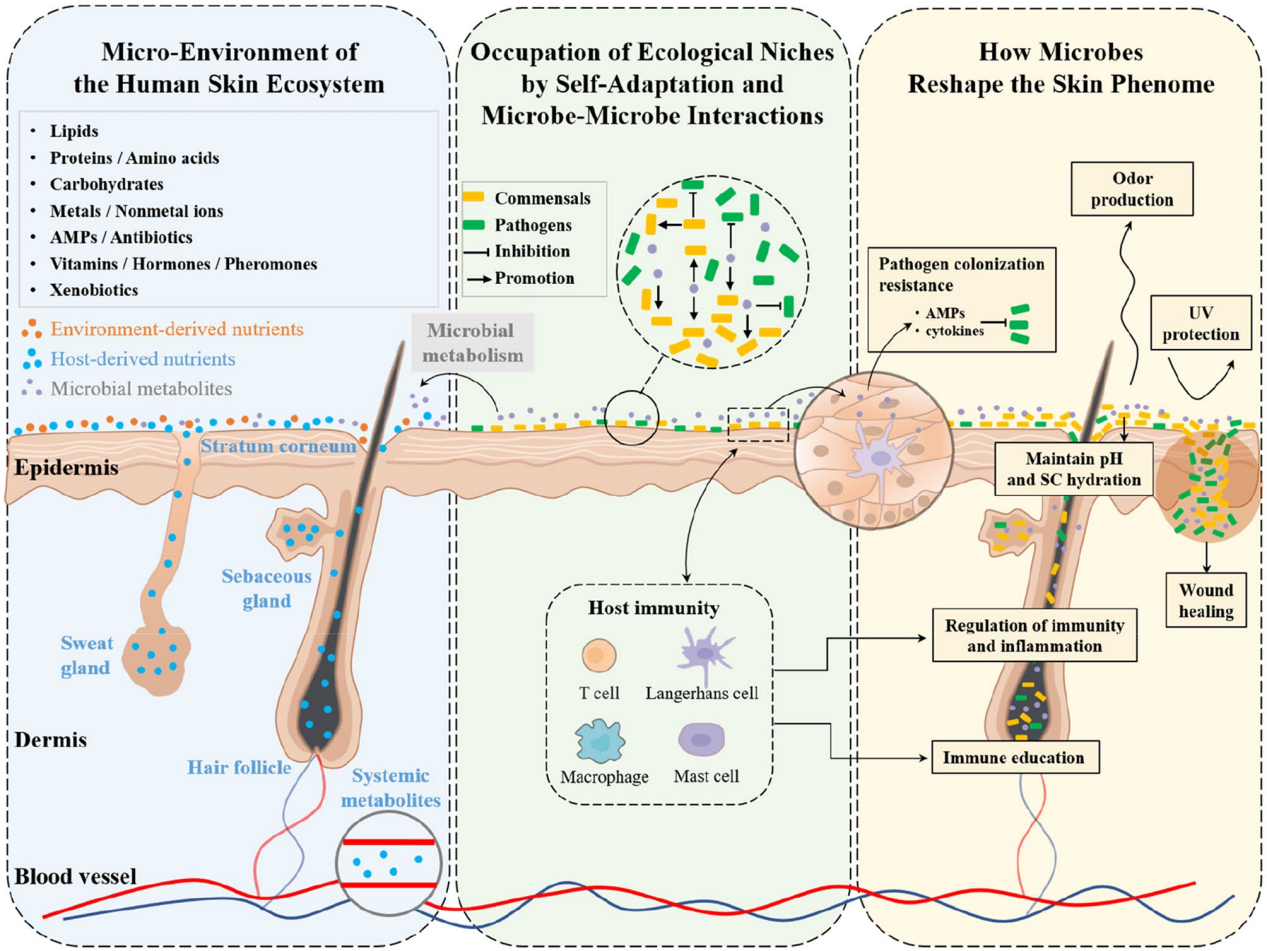
Skin microbiome, metabolome and skin phenome, from the perspective of skin as an ecosystem. From left to right: (Blue box) Diverse substances, derived from the host (stratum corneum, skin appendages, and plasma), environment (xenobiotics) and microbial metabolism, cover the skin surface, forming the micro-environment for skin microbiota; (Green box) occupation of ecological niches by self-adaptation and microbe–microbe interactions, promoting commensals or inhibiting pathogens; (Yellow box) the skin microbes, by their own structures or bioactive molecules, reshape the host skin phenotypes

Profiling the structure of skin microbial community is the first step to overview the ecosystem and to address host–microbe interactions. However, this system was proven to be highly individualized and extremely complex. Many factors were identified influencing the composition of the system, including race, gender, age, lifestyle (e.g., occupation, hygiene, skin product and medication usage, and diet) and environment (e.g., climate, geographical location, pollution, UV, and other radiation) (Wei et al. 2022; Grice and Segre 2011; Harris-Tryon and Grice 2022). Nevertheless, from the perspectives of classical ecology, most of these factors may only indirectly influence, but not drive the establishment and maintenance of the system. The primary selection pressures that form the driving forces for the ecosystem, include resource availability (presence of nutrients), environmental conditions (temperature, geographical access) and biological factors (predators and pathogens) (Williams 1996). In this review, we will sum-up related studies centered on these essential selection pressures, including the presence of different types of nutrients and favored micro-environment for dominant skin commensals, the occupation of the ecological niches through self-adaptation or microbe–microbe interactions, and eventually we will discuss how skin microbes, by their structures or bioactive molecules, reshape host skin phenotypes (Fig. 1).

## Micro-environment of the Human Skin Ecosystem

The host skin offers nutrients and shelters for microbial survival, competition, and cooperation (Roth and James 1988). Nutrient substances may directly affect microbial colonization, growth and metabolism either through nourishing (Brüggemann et al. 2004) or persecuting (Ferrer et al. 2017); on the other hand, these substances may also finetune the local microenvironment, such as pH or moisture state, and thus exert indirect impact on microbial survival. The microbial energy substances are mainly from the host skin and the outside environment. The host skin-derived nutrients consist of lipids embedded in the “brick and mortar” structure (Chen 2018), piles of dead enucleated corneocytes in the stratum corneum (SC) (Abhishek and Palamadai Krishnan 2016), and the secretions from skin appendages [hair follicles (HFs) and glands]. The environment-derived nutrients include personal skincare products, medication, and other environmental xenobiotics. Here, we summarized the metabolites detected on the skin by various metabolome studies (Table1).

**Table 1:** Human skin metabolites: their primary source and functions

Substances	Functions
**Metal and non-metal ions from SC and sweat**	
Sodium, chloride and potassium, calcium, copper, magnesium, zinc, iron, chromium, nickel, lead, manganese, arsenic, mercury, cobalt, molybdenum, strontium, titanium, aluminum, cadmium, lead, nitrogen, iodine, bicarbonate, and phosphorus (Consolazio et al. 1962, 1966; Sears et al. 2012; Minshall et al. 2014; Cohn and Emmett 1978)	Formation of the high-salt environment (Chen et al. 2018) pH of sweat (Sato 1977; Sato and Sato 1990) Regulation of electrolyte homeostasis (Müller et al. 2019) Microbial growth factors (Constante et al. 2017) NMF: potassium, sodium, magnesium, and calcium (Jokura et al. 1995)
**Amino acid and its derivatives from SC and sweat glands**	
l-histidine, threonine, glycine, l-arginine, l-methionine, l-lysine, l-isoleucine, l-leucine, l-valine, l-phenylalanine, tryptophan, l-alanine, l-tyrosine, l-serine, N-acetyl-dl-serine, urocanic acid, uric acid, l-prolinamide, pyroglutamic acid, l-proline, l-carnitine, creatine, l-asparagine, l-glutamine, citrulline, l-glutamate, l-aspartic acid, l-pipecolic acid, ornithine, l-prolinamide, betaine, and taurine (Harshman et al. 2018; Craig et al. 2010)	NMF: l‐serine, Glycine, l‐alanine, histidine, ornithine, citrulline, arginine, and urocanic acid (Caspers et al. 2001; Burke et al. 1966) Skin barrier integrity and appearance (Solano 2020) Acid–base balance and water retention in SC: urocanic acid, serine, and taurine (Solano 2020; Kim et al. 2012, 2021b) Promote wound healing and restore impaired skin: serine, and arginine (Solano 2020; Badiu et al. 2010) UV protection: urocanic acid, phenylalanine, tyrosine, tryptophan, and taurine (Barresi et al. 2011; Wondrak et al. 2006; Kim et al. 2021b) Antioxidant: methionine, tryptophan (Solano 2020; Sardana and Garg 2010) Defense against pathogens: urocanic acid (Solano 2020) Inflammatory and allergic responses: taurine (Solano 2020; Kim et al. 2021b) Collagen synthesis: isoleucine, leucine, and valine (Yamane et al. 2018) Prevention of acne and cold sore: lysine (Solano 2020)
**Peptides, proteins and their derivatives**	
Proteins from SC, viable epidermis and sweat gland	
Urea (Caspers et al. 2001); loricrin (Nithya et al. 2015); keratins (Jokura et al. 1995); filaggrin (Arezki et al. 2017); prolactin-inducible protein, clusterin, apolipoprotein D, PIP (Csősz et al. 2015; Myal et al. 1991); serum albumin, cytokeratin I, Zn-α2-glycoprotein, cystatin A; lipophilin B, CatD (Baechle et al. 2006); protease: several members of the major skin desquamatory family of KLKs (such as KLK1, KLK6-11, KLK13) and cathepsins B, D, Z, F, S, L2, β-chain, MMP8 (Baechle et al. 2006; Yu et al. 2017; Baker 2019)	NMF: filaggrin, urea (Caspers et al. 2001; Arezki et al. 2017) Protect skin from various stresses: keratins, filaggrin, urea, loricrin, apolipoprotein D, and serum albumin (Solano 2020; Nithya et al. 2015; Fluhr et al. 2008; Bajo-Grañeras et al. 2011; Tözsér and Berta 1998) Skin maintenance and protection via desquamation of horny layer, hydrolysis of debris in the ductal lumen, allergen inhibition: proteolytic enzymes (Yokozeki et al. 1991) Tissue regeneration: apolipoprotein D (Bajo-Grañeras et al. 2011) Transport, binding, antioxidant and catalytic activity role: serum albumin, protease (Yu et al. 2017; Gum et al. 2004) Immunological functions: Prolactin-inducible protein bind to IgG, IgG-Fc, CD4-T cell receptor (Autiero et al. 1991; Lee et al. 2002) and also to different species of bacteria such as *streptococci* (Nistor et al. 2009; Hassan et al. 2009) Chaperone, modulator of MMP9 activity: clusterin (Schenkels et al. 1997; Jeong et al. 2012)
Neuropeptides from sweat gland	
SP, CGRP (N'Diaye et al. 2017)	Sense microbes and critical for skin homeostasis (N'Diaye et al. 2017) Modulator of skin microbiome virulence (N'Diaye et al. 2017) Anti-inflammation (Choi et al. 2018): low concentrations of SP
Antimicrobial peptides (AMPs) from sweat, sebocytes and keratinocytes (KCs)	
RNAse7, S100 proteins (S100A7, S100A8, S100A9, S100A12 and S100A15), hBD-1-3, cathelicidins (Büchau and Gallo 2007); active form of cathelicidin (NL-8, LR-10, KR-10, IK-14, LL-17, LL-23, KR-20, KS-27, KS-30, and LL-37) (Yamasaki et al. 2006; Murakami et al. 2002); DCD (Lousada et al. 2021; Reithmayer et al. 2009); DCD-1L and DCD-1L derived peptides (Schittek et al. 2001); cathelicidin hCAP-18 (Sørensen et al. 2001; Baechle et al. 2006); histone H4 (Lee et al. 2009); LF (Park et al. 2011); sIgA (Imayama et al. 1994); Lcn2 (Takahashi and Yamasaki 2020)	Participation in epithelial innate defense and defense against pathogens (Serag et al. 2021; Gläser et al. 2005; Nizet et al. 2001; Park et al. 2011) Enhance the antimicrobial action of FFAs in human sebum: histone H4 (Lee et al. 2009)
Cytokines/chemokines/antibodies from KCs and sweat	
IL-1α, 1β, 6, 8, 25, 31, 36, TNF-α, IFN-β and CXCL10, IgG, IgA (Takahashi and Yamasaki 2020; Dai et al. 2013; Baker 2019)	Prime and amplify epidermal innate immune signals with the dermal adaptive immune system (Takahashi and Yamasaki 2020; Li et al. 2018b; Xu et al. 2018) Defense against pathogens (Baker 2019; Li et al. 2018b)
**Sugar from sweat, cosmetics and extracellular matrix**	
Lactate (Caspers et al. 2001); glucose, fructose, mannose, and galactose (Roux et al. 2022); *β*-glucans (Du et al. 2014); hyaluronic acid (Lew and Liong 2013)	NMF: lactate (Caspers et al. 2001) The elevated glucose level promotes itching and delay the recovery of skin barrier (Ono et al. 2018) Anti-wrinkle, wound healing, antioxidant activity, anti-UV effect, and moisturizing effect: *β*-Glucans (Du et al. 2014) Epidermal barrier regulation: hyaluronic acid (Lew and Liong 2013) Enhance self-defense of the skin for infection: low molecular weight hyaluronic acid (Gariboldi et al. 2008)
**Lipid and its metabolites**	
Sweat-derived lipids	
Over 150 lipid mediators, including prostanoids, alcohols, diols, epoxides, ketones, nitrolipids, N-acylethanolamides, monoacylglycerols, and ceramides (Agrawal et al. 2018); lauric acid (C12:0), myristic acid (C14:0), palmitic acid (C16:0), oleic acid (C18:1), and stearic acid (C18:0) (Nunome et al. 2010); lactic acid; pyrrolidone-5-carboxylic acid (Caspers et al. 2001); 5-aminopentanoic acid, and l-pipecolic acid (Harshman et al. 2018)	Extracellular stimuli response: lipid mediators (Murakami 2011) Antimicrobial, anti-inflammatory effect: lauric acid, oleic acid, and lactic acid (Drake et al. 2008; Fischer et al. 2012; Clayton et al. 2019; Lew and Liong 2013) NMF: lactic acid, pyrrolidone-5-carboxylic acid (McGrath 2008; Caspers et al. 2001)
Epidermal (SC) Lipids	
Ceramides (Unique to epidermis) (Pappas 2009); FAs: saturated FFAs, monounsaturated FAs, polyunsaturated FAs (PUFAs), and hydroxyl FFAs (Ansari et al. 1970); cholesterol (Cui et al. 2016)	Barrier against the chemical, physical, and microorganism insults (Feingold 2009)
Sebaceous lipids from sebum (sebaceous glands)	
TG and FAs (Greene et al. 1970); diglycerides, wax esters (Pappas 2009); squalene (Thiboutot 2004; Nicolaides 1974; Thody and Shuster 1989); cholesterol, cholesterol esters (Greene et al. 1970); sapienic acid (C16:1Δ6) (Pappas 2009; Nicolaides 1974); sebaleic acid (18:2Δ5, 8) (Picardo et al. 2009); oleic acid (18:1Δ9) (Lovászi et al. 2017)	Maintain skin surface moisture permeability: wax ester, FFAs, and squalene (Cui et al. 2016; Pappas 2009) Antimicrobial, antioxidant, anti-inflammatory effect: squalene, wax esters, FFAs, cholesterol ester, sapienic acid, and oleic acid (Nakatsuji et al. 2010; Pappas 2009; Kim and Karadeniz 2012; Cui et al. 2016) Mediate immune responses: FFAs (Cui et al. 2016) UV protection: squalene (Ohsawa et al. 1984)
Plasma lipids	
Cholesterol, plant sterols, β-sitosterol, campesterol, and stigmasterol (Bhattacharyya et al. 1972); lathosterol and lanosterol (Bhattacharyya et al. 1972); itaconic acid, crotonic acid and heptadecanoic acid, xanthine, d-ribose 5-phosphate, and uric acid (Chen et al. 2021)	Participation in lipid metabolism: itaconic acid, crotonic acid, and heptadecanoic acid (Chen et al. 2021) Positive correlated with specific skin bacteria: itaconic acid, crotonic acid, and heptadecanoic acid (Chen et al. 2021) Participation in nucleotide metabolism: xanthine, d-ribose 5-phosphate, and uric acid (Chen et al. 2021)
Lipids in cosmetic products/personal care products	
* o*-formylbenzoic acid, oleic acid, palmitic acid, and monoacylated glycerols monoolein and monopalmitin (Bouslimani et al. 2015); mineral oils and waxes (Petry et al. 2017)	Shaping the chemical environment for specific skin microbial communities (Bouslimani et al. 2019) Provide nutrients and promote the growth of lipophilic bacteria (Bouslimani et al. 2015; Unno et al. 2017; Holland et al. 2010)
**Vitamins mainly from sweat**	
Niacin (Sargent et al. 1944); vitamin D (Cornbleet et al. 1936; Lugg and Ellis 1954; Dam 1978; van der Beek 1991); l-ascorbic acid (Vitamin C) (Harshman et al. 2018); vitamin E (Cornbleet et al. 1936; Lugg and Ellis 1954; Dam 1978; van der Beek 1991); niacinamide (Gehring 2004)	Maintenance of epidermal barrier and moisture: niacinamide (Gehring 2004) Anti-inflammatory, anti-aging effect: niacinamide, vitamin C, and vitamin E (Cornbleet et al. 1936; Lugg and Ellis 1954; Dam 1978; van der Beek 1991; Gehring 2004) UV protection: active vitamin D3, and vitamin C (Pullar et al. 2017; Bocheva et al. 2021)
**Pheromones from sweat glands and sebaceous glands**	
Releaser/primer/signaler/modulator pheromones (Preti et al. 2003); adrenal glucocorticoids (Nichols and Miller 1948)	Body odor (Baker 2019) Generate immediate, primarily behavioral responses: releaser pheromones (Preti et al. 2003) Generate slower physiological/endocrine/neuroendocrine responses: primer pheromones (Preti et al. 2003) Mood and multisensory inputs regulation: modulator pheromones (Jacob and McClintock 2000)
**Other xenobiotics from the environment, i.e. pollutants or personal care products**	
PAHs (Leung et al. 2020) POPs (organochlorinated pesticides, polychlorinated biphenyls, perfluorinated compounds) and other toxicants (BPA, heavy metals, phthalate, and polybrominated diphenyl ethers) (Baker 2019) Drugs (griseofulvin, ketoconazole, beta-lactam antibiotics, ceftazidime, ceftriaxone and isotretinoin) (Hoiby et al. 2000; Sato et al. 1989a, 1989b; Tilles 2014) Cosmetics (preservatives, moisturizers, foundation, foot powder, deodorant, topical prebiotics, and topical postbiotics) (Salminen et al. 2021; Pinto et al. 2021; Murphy et al. 2021) Others (e.g., ethanol, pyrrolidine, piperidine, trolamine, and diolamine) (Harshman et al. 2018)	Influence the function and structure of skin microbiome: PAHs (Leung et al. 2020) Promote premature skin aging, pigmentary disorder, acne, and skin cancer: PAHs (Leung et al. 2020) Cause vitamin D deficiency: POPs (Bocheva et al. 2021) Antibiotics increased antibiotic resistance: drugs Modulation of dihydrotestosterone formation: isotretinoin (Tilles 2014) Cosmetics Influence the function and structure of skin microbiome: foundation and foot powder (Bouslimani et al. 2015, 2019; Elpa et al. 2021; Staudinger et al. 2011; Boxberger et al. 2021) Favor the growth of potential pathogens, such as *S. aureus*: emulsifiers (Krogsgård Nielsen et al. 2016) Provide nutrients and promote the growth of lipophilic bacteria such as *Staphylococcus* and *Propionibacterium*: moisturizers (Bouslimani et al. 2015; Unno et al. 2017; Holland et al. 2010) Preservatives exert antimicrobial effect in vitro (Pinto et al. 2021; Wang et al. 2019a; Murphy et al. 2021), such as inhibit the growth and biofilm formation of *S. aureus* or pathogenic *C. acnes *in vitro (Gannesen et al. 2019), but no influence on the skin microbiome in vivo (Murphy et al. 2021)

*NMF* natural moisturizing factor, *PIP* prolactin inducible protein, *CatD* cathepsin D, *KLKs* kallikrein-related peptidases, *MMP* matrix metalloproteinase, *SP* substance P, *CGRP* calcitonin gene-related peptide, *DCD* dermcidin, *hBD* human β-defensins, *LF* Lactoferrin, *sIgA* Secretory form of immunoglobulin A, *S100A7* psoriasin, *S100A8* calgranulin A, *S100A9* calgranulin B, *S100A12* calgranulin C, *Lcn2* lipocalin-2, *IL* Interleukin, *TNF-α* tumor necrosis factor-α, *IFN-β* interferon-beta, *TG* triglyceride, *FAs* fatty acids, *FFAs* free fatty acids, *PAHs* polycyclic aromatic hydrocarbons, *POPs* persistent organic pollutants, *BPA* bisphenol-A

It is known that individuals, even the same individual at different life stages, vary markedly in regards to the delicate structure or secretion function of the skin and appendages, which produce metabolites consistently and thus play an essential role in shaping diverse microenvironments with distinct pH, salt, moisture, sebum content, and extent of anaerobiosis (Grice and Segre 2011; Capone et al. 2011; Grice et al. 2009). Factors that influence systemic metabolisms, such as diet and gut microbiota, and hormone levels, can also significantly impact the skin’s local microhabitats (Prescott et al. 2017). Furthermore, one’s exposome, such as environmental pollution, UV levels, occupation environment, drug or skincare habits, is highly individualized (Khmaladze et al. 2020). These together form highly complex physical and chemical landscapes on the skin surface, likely to be the real biological explanation that underlies the substantial inter-individual variability in the skin microbiota. Indeed, our previous study showed two robust “cutotypes” of microbial networks on Chinese facial skin, *C-cutotype* and *M-cutotype,* possessed distinct patterns of skin properties (Li et al. 2021). The dominant two species, *C. acnes* and *Moraxella osloensis,* exhibited vastly varied nutrient-demand: whereas *C. acnes* was high nutrient demanding, *M. osloensis* was a non-fastidious bacterium that was able to grow in a mineral medium supplemented with a single organic carbon source (Juni 1974; Juni and Bøvre 2015). This species was shown to be incapable of utilizing any carbohydrates or possessing any saccharolytic activity, but strictly depend on other carbon sources such as acetic or lactic acid (Baumann et al. 1968; Juni 1974; Juni and Bøvre 2015; Moss et al. 1988).

## Occupation of Ecological Niches by Self-adaptation and Microbe–Microbe Interactions

The skin surface formed diverse microhabitats, and many studies favored to divide them into four types (sebaceous, moist, dry, and foot) according to the physical properties of anatomical locations (Oh et al. 2014). Although such water/oil-based classification was not delicate enough, some prominent features for the growth and colonization of the microbiota were well identified. Other metabolites and physical properties were also identified in modulating microbial communities. Furthermore, microbe–microbe interactions are essential for shaping the skin ecosystem. In general, microbes deploy strategies to adapt to the living environment and compete for ecological niches via the following: (1) Self-adaptation to the specific environment conditions: skin microbiota changes their characteristic like metabolism pathways to adapt to the skin microenvironment. For example, *Staphylococcus* synthesized tensioactive agent to withstand the low pH and high salt content of sweat (Hentati et al. 2021; Scharschmidt and Fischbach 2013); (2) Competition for ecological niches through microbe–microbe interactions, for example, coagulase-negative Staphylococcus (CoNS) species can either directly kill or limit the virulence of *Staphylococcus aureus* through the secretion of different regulators (Flowers and Grice 2020). Here we will sum-up the findings of this part (Table 2).

**Table 2:** Features of dominant skin commensals for the occupation of ecological niches

Favorable microenvironment	Biology basis for self-adaptation	Occupation of ecological niches by microbe–microbe interactions
***Cutibacterium*** **(gram-positive anaerobic bacilli)** *C. acnes*, *C. granulosum*, and *C. avidum*		
HFs with low oxygen content (Scharschmidt and Fischbach 2013) Sebum-rich areas, i.e. the face, scalp, chest, and back (Scharschmidt and Fischbach 2013; Brown and Shalita 1998) Moist areas: *C. avidum* (McGinley et al. 1978)	*C. acnes* Utilize nutrients from SC, sebum, and sweat (Scharschmidt and Fischbach 2013) by secreting lipase (Brown and Shalita 1998; Brüggemann et al. 2004) and proteases (Holland et al. 1979) Catabolize sebum to FFAs for better skin attachment (Brüggemann et al. 2004; Brown and Shalita 1998; Miskin et al. 1997; Gribbon et al. 1993) Secrete porphyrins to oxidize squalene and lower oxygen tension in HFs (Tilles 2014; Holland et al. 1998)	*C. acnes* Secrete propionicin to defend against Gram-positive and Gram-negative anaerobes (Christensen and Bruggemann 2014) Secrete RoxP to facilitate the growth of aerobic bacteria (Allhorn et al. 2016) Produce FFAs to acidify the skin to inhibit colonization by other pathogenic microbes (*S. aureus* and *Streptococcus pyogenes*) (Youn et al. 2013) Produce coproporphyrin III to induce *S. aureus* aggregation and biofilm formation (Wollenberg et al. 2014) Produce CAMP factor to intensify the virulence of *S. aureus* (Lo et al. 2011) Produce a thiopeptide antibiotic, cutimycin, to limit S. aureus colonization (Claesen et al. 2020)
***Staphylococci*** **(gram-positive cocci aerobes or facultative anaerobes)** CoNS: *S. epidermidis*, *S. capitis*, *S. caprae*, *S. hominis*, *S. lugdunensis*, and *S. haemolyticus*		
Highly adaptable: occlude areas (axilla), exposed dry sites (volar forearm) and also low oxygen area of the HFs (Scharschmidt and Fischbach 2013) * S. epidermidis* favors areas of high eccrine glands density, high moisture, temperature and pH (Scharschmidt and Fischbach 2013) Nasal: *S. lugdunensis* (Zipperer et al. 2016; Nakatsuji et al. 2017)	*Staphylococci* are able to utilize diverse nutrients from SC, sebum and sweat (Scharschmidt and Fischbach 2013) *Staphylococcus* can synthesize tensioactive agents to withstand the low pH and high salt content of sweat (Hentati et al. 2021; Scharschmidt and Fischbach 2013) *S. epidermidis* High-salt tolerance (Scharschmidt and Fischbach 2013) Possess various adhesins for colonization (Ginsburg 2002; Scharschmidt and Fischbach 2013; Flowers and Grice 2020) Produce enzymes for esterifying FAs that protect from abundant bactericidal lipids (Chamberlain and Brueggemann 1997)	*S. epidermidis*, *S. hominis* and *S. capitis* secrete lantibiotics, class II bacteriocins, PSMs or AMPs to inhibit MRSA, *Streptococcus pyogenes*, *S. aureus* and *C. acnes*, and synergize with the human AMP LL-37 to enhance skin defense (Nakatsuji et al. 2017; Bastos et al. 2009; Cogen et al. 2010; O'Neill et al. 2020; Janek et al. 2016) *S. epidermidis* secrete 6-HAP or SCFAs to inhibit GAS, MRSA and *S. aureus* growth (Nakatsuji et al. 2018; Wang et al. 2014; Keshari et al. 2019; Kao et al. 2017) *S. epidermidis* produce and release Esp to inhibit biofilm formation and disrupt the biofilm of *S. aureus* (Iwase et al. 2010) *S. lugdunensis* secrete lugdunin to inhibit *S. aureus* (Zipperer et al. 2016) *S. capitis* antagonize *S. aureus* through interference with the agr quorum sensing pathways, which are required for S. aureus virulence (Paharik et al. 2017; Williams et al. 2019)
* S. aureus* (coagulase-positive)		
Moist skin sites (nasal, axillary, inguinal and rectal areas) (Kluytmans et al. 1997; Yang et al. 2010)	Form biofilm (van Loosdrecht et al. 1990) Multi-drug resistance (Wang et al. 2019b)	Opportunistic pathogen Acquire ACME horizontally from S. epidermidis to optimize growth conditions for nutrients and survival (Diep et al. 2006; Scharschmidt and Fischbach 2013)
***Corynebacteria*** **(gram-positive aerobes or facultative anaerobes belonging to the Phylum Actinobacteria)** *C. accolens*, *C. jeikeium, C. urealyticum, C. amycolatum, C. minutissimum, C. striatum*, and *C. pseudodiphtheriticum*		
Moist and sebaceous skin sites (Scharschmidt and Fischbach 2013) Occluded areas (Flowers and Grice 2020) Nasal cavity: *C. pseudodiphtheriticum*, *C. accolens* (Hardy et al. 2019)	Acquire nutrients from SC, sebum and sweat, depending on lipase (Scharschmidt and Fischbach 2013; Houpt 2005; Flowers and Grice 2020) Halotolerant (high-salt) (Scharschmidt and Fischbach 2013) Generate mycolic acid layer to resist multiple stresses, such as detergents, antimicrobials, and lysozyme, allowing colonization across various conditions (Burkovski 2018; Tauch and Burkovski 2015) *C. striatum*: multi-drug resistance (Wang et al. 2019b)	*C. accolens* produce FFAs to inhibit *S. pneumoniae* (Bomar et al. 2016) *C. striatum* modulate the Agr quorum-sensing system and expression of Agr-inducible virulence genes to limit *S. aureus* (Ramsey et al. 2016) *C. pseudodiphtheriticum* mediate bactericidal activity against *S. aureus* (Hardy et al. 2019)
**Fungi** *Malassezia*: *M. dermatis*, *M. furfur*, *M. globosa*, *M. restricta*, and *M. sympodialis*		
Relatively stable at different sites (Bouslimani et al. 2019; Findley et al. 2013) *Malassezia* favored lipid-rich areas, such as the face, scalp, back and outer ears (Kaneko et al. 2010) *M. sympodialis* (nares, antecubital crease, volar forearm, and hypothenar palm); *M. globose* (back, occiput, and inguinal crease); *M. restricta* (external auditory canal, retroauricular crease, and glabella) (Findley et al. 2013); *M. obtuse* (groin, nasal vestibule) (Grice and Dawson 2017)	*Malassezia* enrich glycosyl hydrolases and genes involved in carbohydrate metabolism, concordant with adaptation to a carbohydrate-deficient and lipid-rich environment (Wu et al. 2015) *Malassezia* acquired a catalase horizontally to protect *Malassezia* cells from their own secreted hydrogen peroxide generating proteins (Wu et al. 2015) *Malassezia* aquired flavohemoglobins horizontally from the bacterial genus *Corynebacterium*, increasing NO resistance (Ianiri et al. 2020; Wisecaver et al. 2016)	*M. globosa* secrete protease (MgSAP1) to degrade virulence protein of *S. aureus* and inhibit its biofilm formation (Li et al. 2018a; Ianiri et al. 2018) *Malassezia* produce VOCs to inhibit *S. aureus*, *Bacillus subtilis* and *Escherichia coli* (Al-Fatimi et al. 2016) *M. sympodialis, M. globosa, and M. slooffiae* can form biofilms to be potential pathogens in community (Angiolella et al. 2020)
**Others** **Fungi**: *Aspergillus, Cryptococcus, Rhodotorula, Epicoccum,* and others (Findley et al. 2013) **Probiotics**: *Enterococcus faecalis* SL-5, *Lactobacillus*, *Bifidobacteria,* and *Nitrosomonas eutropha* (Kang et al. 2009; Lew et al. 2013; Lee et al. 2018; Notay et al. 2020)		

*Roxp* Radical oxygenase of Propionibacterium acnes, *CAMP* Christie, Atkins, Munch Peterson, *PSMs* Phenol-soluble modulins, *6-HAP* 6-N-hydroxyaminopurine, *SCFAs* Short-chain fatty acids, *GAS* group A Streptococcus, *MRSA* Methicillin-resistant Staphylococcus aureus, *Esp* Serine protease, *ACME* Arginine catabolic mobile element, *Agr* Accessory gene regulator, *MgSAP1* Malassezia globosa Secreted Aspartyl Protease 1, *VOCs* volatile organic compounds

Compared to the skin surface, HFs provide a more moisture and acidic environment with ultraviolet light protection, facilitating the colonization of multiple bacteria, fungi, and viruses. The most abundant bacteria in the HFs were *P. acnes* spp. (Lousada et al. 2021)*. M. restricta* and *M. globosa* are the dominant fungi (Lousada et al. 2021). Meanwhile, the HF virome comprises dependoviruses, *Propionibacterium* phage P100D and 101A, papillomaviruses and adeno-associated viruses (Hall et al. 2018). In addition, the mite (*Demodex folliculorum*) groups are often found in the distal infundibulum, usually with their dorsal body oriented against the hair shaft (Elston and Elston 2014).

## From Microbes to Host Skin: How Microbes Reshape the Skin Phenome

Skin microbiota leverage “nutrients” from the host skin and environment and produce a series of bioactive molecules with vital functions (Chen et al. 2018). For example, skin microbiota can convert host proteins into amino acids by their protease (Holland et al. 1979; Byrd et al. 2018), ferment carbohydrates into lactic acids (Ong et al. 2020) or decompose sebum lipids such as triglycerides into free fatty acids (FFAs) (Traisaeng et al. 2019; Belkaid and Segre 2014). In addition, skin microbiota produces AMPs, phenol-soluble modulins (PSMs), and antibiotics (Belkaid and Segre 2014; Gallo and Hooper 2012). These metabolism products may further act on the host or other microbes, exert biological effects and reshape the skin phenome.

The most well-studied functions of skin commensals include the following: (1) pathogen colonization resistance by ecological niche blocking for the invasion of opportunistic or pathogenic microbiota, (2) immune education during early phases, and (3) regulation of immunity and inflammation. Given many comprehensive reviews already on these functions, we will take a particular focus on other functions that were usually missed, including the maintenance of skin physiology, such as pH and SC hydration, UV protection, odor production, and wound healing, which were also important functions in skin homeostasis.

### Regulation of Immunity and Inflammation

The microbiota is a rich source of short-chain fatty acids (SCFAs) (Traisaeng et al. 2019). For example, *C. acnes* fermented carbohydrates into propionic acid (Traisaeng et al. 2019); *S. epidermidis* was able to ferment glycerol to butyric acid and acetic acid in vitro (Traisaeng et al. 2019; Keshari et al. 2019). SCFAs can regulate several immune cell functions, including the production of cytokines (TNF-α, IL-2, IL-6, and IL-10) (Traisaeng et al. 2019), activate resident skin regulatory T (Treg) cells, mitigate inflammatory skin reactions and thus contribute to the preservation of skin homeostasis in mice and human (Schwarz et al. 2017). Butyric acid significantly attenuated lipopolysaccharide (LPS)-induced nuclear factor-κB (NF-κB) activation and nitric oxide production in murine macrophage cell line (Chakravortty et al. 2000), reduced interferon-gamma (IFNγ)-induced proinflammatory IL-6 and TNF-α production in a macrophage cell line (Park et al. 2007) and mediated short-chain fatty acid receptor 2 (FFAR2) to modulate the production of proinflammatory cytokines induced by ultraviolet B (UVB) in mice (Keshari et al. 2019). Furthermore, the ability of immune cells to migrate to the foci of infection can be regulated by SCFAs (Vinolo et al. 2011). Given the potential anti-inflammatory of SCFAs, they are applied on psoriatic skin in vitro. This study found that decreased expression of G-protein-coupled receptors (GPR) GPR43 and GPR109a in psoriatic skin can be restored and expression of inflammatory factors can be inhibited by topical application of sodium butyrate (Krejner et al. 2018). However, SCFAs are not always anti-inflammatory. *C. acnes*-derived SCFAs inhibit histone deacetylase (HDAC) activity in skin keratinocytes (KCs) and stimulate inflammation through Toll-like receptor (TLR) signaling (Sanford et al. 2016). SCFAs from *C. acnes* conferred a robust proinflammatory effect in human sebocytes (Sanford et al. 2019). Expression of a major component of the *Corynebacterium accolens* cell wall, mycolic acid, promotes inflammation in an IL-23-dependent manner under a high-fat diet condition in mice (Ridaura et al. 2018).

The essential amino acid tryptophan (Trp) can be metabolized by human skin microbiota into 5-hydroxytryptophan (5-HTP), indole-3-aldehyde (IAId) and other metabolites (Yu et al. 2019). IAId was able to suppress thymic stromal lymphopoietin (TSLP) and thereby inhibited calcipotriol (MC903)-induced AD-like dermatitis in mice (Yu et al. 2019). IAId can also activate aryl hydrocarbon receptor (AhR), producing indoleamine 2,3-dioxygenase (IDO) and IL-10 in Langerhans cells (LCs), and thus negatively regulate skin inflammation (Liu et al. 2020).

*S. epidermidis* and other Gram-positive bacteria release adhesion molecules upon bacteriolysis, such as lipoteichoic acid (LTA) (Ginsburg 2002). LTA from *Staphylococcal* species suppressed inflammation during tissue injury through a Toll-like receptor 2 (TLR2)-dependent mechanism to prevent excessive damage (Lai et al. 2009). Staphylococcal LTA may also have applications in the treatment of inflammatory disease. For example, in an acne model of *C. acnes*-induced skin inflammation, staphylococcal LTA application abrogated inflammatory effects via induction of a microRNA, miR-143, destabilizes the TLR2 mRNA and decreases protein production (Xia et al. 2016).

In addition, many commensal species contain virulence strains. One major virulence factor of the microorganism is a secretory lipase that acts on triglycerides to release FFAs (Holland et al. 2010). *C. acnes* exist both in health and patients, but *C. acnes* from acne patients harbored unique genomic elements encoding virulence factors, including camp5, gehA, sialidases, neuraminidases, endoglicoceraminidases, lipases, proteases and hemolysins that were rarely present in *C. acnes* genomes from healthy controls (Brüggemann 2005; Burkhart et al. 1999). Several commensals are opportunistic pathogens that encode virulence factors such as toxins, exoenzymes, and adhesins (Brown et al. 2012). Skin microbiota may directly or indirectly mediate inflammatory responses by releasing various virulence factors under unhealthy conditions. *Malassezia spp.* can be the causative agents in disease. Many Ma*lassezia spp.* secrete extracellular vesicles that signal KCs to secrete proinflammatory cytokines (Vallhov et al. 2020; Watanabe et al. 2001; Zhang et al. 2019). *Malassezia spp.* metabolize sebum to different fatty acids such as phosphatidylcholine (PC) and phosphatidylserine (PS), which then act as irritants, causing flaking and irritation under dandruff, a frequent scalp issue and seborrheic dermatitis conditions (Celis Ramírez et al. 2020; DeAngelis et al. 2005; Han et al. 2019; Johansson et al. 2018).

### Pathogen Colonization Resistance

Commensals compete for niches through microbe–microbe interactions, as mentioned above (Table 2). Direct induction of AMPs or cytokine expression in KCs is one of the main strategies used by skin commensals, such as *Propionibacterium* and *S. epidermidis*, in defending against pathogen invasion and shaping the skin microbiota community (Midorikawa et al. 2003; Wanke et al. 2011). In addition, commensals function as endogenous cofactors of the skin immune system to promote skin local immune response. Skin harbor considerable commensal-specific T-cell, e.g., *Staphylococcus epidermidis*-specific IL-17A^+^ CD8^+^ T cells (Naik et al. 2015). The activation of these cells can promote AMP production by keratinocytes, thereby promoting heterologous protection against pathogens infections (Braff et al. 2005). *Staphylococcus epidermidis* can also induce KC to express IL-1α, thus promoting skin αβ T cells to produce IL-17A and IFNγ in mice (Naik et al. 2012). IL-17A induces chemokines that recruit neutrophils and AMP production, thus protecting the host from pathogen infection. In adults, cutaneous mucosal-associated invariant T cells (MAIT cells) are a dominant population of IL-17A-producing lymphocytes (Constantinides et al. 2019). MAIT cells are absent in germ-free (GF) mice, and their development are controlled by microbial metabolites such as vitamin B2 (Treiner et al. 2003; Koay et al. 2016; Legoux et al. 2019). MAIT cells can respond to skin commensals or commensal-derived metabolites in an IL-1-, IL-18-, and antigen-dependent manner (Constantinides et al. 2019), thus enhancing inhibition of pathogen invasion.

### Immune Education

The commensals play an essential role in regulating the development, proliferation, maturation and activation of immune cells of innate immunity. A previous study found that GF mice contain mast cells (MCs) that are largely undifferentiated and express abnormally low amounts of stem cell factor (SCF). Commensal bacteria induce KC-produced SCF, promote skin MCs mature. The migration of MCs in the skin is fully dependent on high levels of SCF, as produced by KCs (Wang et al. 2017b). In addition, γδT cells, which play an essential role in recognizing lipids, one of the microbial metabolites (Belkaid and Tamoutounour 2016), significantly reduced IL-17 secretion capacity in GF mice (Naik et al. 2012). Varying from the immune responses to invasive pathogens, adaptive immune responses respond to commensals under noninflammatory conditions, which help build immune homeostasis (Naik et al. 2015).

The skin contains one of the highest frequencies of FOXP3^+^ Treg cells within the body in mice (Suffia et al. 2006). In the skin of both mice and humans, Tregs reside in the dermis, and a large fraction of these cells can be found in close proximity to HFs, which serve as a natural habitat for skin-resident microorganisms (Ali et al. 2017; Sanchez Rodriguez et al. 2014). Tregs are essential in establishing and regulating immune tolerance to commensal microbes during a defined period of neonatal life in mice (Scharschmidt et al. 2015). *S. epidermidis* colonization on the skin surface two weeks after birth induces Treg cells’ tolerance to *S. epidermidis* in adult mice (Scharschmidt et al. 2015). Furthermore, it promotes the accumulation and migration of Treg cells into the skin (Scharschmidt et al. 2017). Further study found that Treg cell migration in Neonatal Skin is influenced by hair follicle development and microbes colonized in the hair follicle. In turn, colonization of microbes in HFs during the early stage is resisted and regulated by Treg cells (Scharschmidt et al. 2017). These results suggest a dynamic balance between microbe and host immune system.

### Maintain pH and SC Hydration

Skin microbiota metabolizes dead corneocytes, sweat and sebum components, and other wastes (Pistone et al. 2021) and converts them into amino acids, such as glutamate and aspartate, proteins and various FFAs (Pistone et al. 2021; Timm et al. 2020). They also secrete lactic acid (Ong et al. 2020), a series of SCFAs (Christensen and Brüggemann 2014) and other organic acids (Garrote et al. 2000; Wang et al. 2017a; Bengoa et al. 2019). These acidic metabolites can regulate skin surface pH and SC hydration level (Watabe et al. 2013; McGrath 2008; Caspers et al. 2001; Cui et al. 2016; Pappas 2009).

The skin surface pH is slightly acidic, ranging from 4.5 to 5.5 in human (Braun-Falco and Korting 1986). The pH of the SC is crucial for many vital epidermal functions, including permeability barrier homeostasis, desquamation of corneocytes, initiation of inflammation, processing of secreted lamellar body (LB) polar lipids and antimicrobial defense (Lee and Lee 2014). In addition, variation in pH also affects the SC thickness and pigmentation (Sandby-Møller et al. 2003). These results indicate that many skin traits may intertwine, such as pH, trans-epidermal water loss (TEWL), skin thickness, SC hydration and pigmentation, and thereby may be modulated by skin microbiota and their metabolites.

Our previous study also revealed that cutotypes of microbial networks on Chinese facial skin possess distinct skin traits: *C*-*cutotype* skin is more hydrated and more oily, and the levels of skin surface sebum and its microbial metabolite porphyrin are increased; In contrast, *M-cutotype* skin is dryer and often occurs in the elder (Li et al. 2021). A study on the skin microbiome of Koreans found that *Lawsonella* had a negative correlation with skin moisture and brown spots; *Staphylococcus* and *Corynebacterium* both had negative correlations with the number of UV spots and positive correlations with TEWL; *Staphylococcus aureus* had a negative correlation with skin moisture parameters (Kim et al. 2021a). Moreover, two studies found a linkage between the skin microbiome and skin metabolites (Howard et al. 2022; Roux et al. 2022). A recent study demonstrated that *S. epidermidis* can significantly increase skin ceramide levels and thereby prevent water loss of damaged skin dependent on its sphingomyelinase in mice (Zheng et al. 2022).

Skin aging is a dynamic process with a series of changes in the skin phenome (Farage et al. 2008; Pochi et al. 1979; Cotterill et al. 1972; Howard et al. 2022) and skin metabolism, e.g., altered levels of natural moisturizing factors (NMFs), AMPs, vitamins and coenzyme Q10, and many other metabolites (Howard et al. 2022; MacLaughlin and Holick 1985; Kuehne et al. 2017). These changes may underlie the alterations in the microbiome. For example, age-related decrease in sebocyte area is positively correlated with *Cutibacterium* and negatively correlated with *Streptococcus*, *Acinetobacter*, *Corynebacterium* and *Methylobacterium‒Methylorubrum* abundance (Howard et al. 2022). Furthermore, anti-aging skincare products were reported able to persist on the skin for weeks and provide long-term contributions to the chemical environment (Bouslimani et al. 2019), thus shaping the specific skin microbial communities (Bouslimani et al. 2015). For example, lipid components of moisturizers could provide nutrients and promote the growth of lipophilic bacteria such as *Staphylococcus* and *Propionibacterium* (Bouslimani et al. 2015; Unno et al. 2017; Holland et al. 2010). More details regarding cosmetics can be found in Table 1.

### UV Protection

Some skin commensals can protect skin from UV damage by secreting different metabolites (Souak et al. 2021). For example, *S. epidermidis* can produce 6-HAP to suppress UV-induced tumor in mice (Nakatsuji et al. 2018). Skin microflora produces *cis*-urocanic acid from l-histidine, affects UV-induced immune suppression and suppresses melanoma growth (Hug et al. 1999; Laihia et al. 2010). Some *Streptomyces*-derived compounds, such as amides exhibited UV-absorbing, antioxidant, and anti-inflammatory properties (Sánchez-Suárez et al. 2020). Propionic acid produced by *Cutibacterium acnes* fermentation ameliorates UVB-induced melanin synthesis (Kao et al. 2021). *Cyanobacteria* develop a diversity of defense mechanisms, including the biosynthesis of UV-absorbing/screening compounds, such as mycosporine-like amino acids (MAAs), and enzymes, including superoxide dismutases (SOD), which counteract oxidative stress (Souak et al. 2021).

Ultraviolet radiation (UV-R) is well known to inhibit the cellular growth of *Malassezia furfur* (Wikler et al. 1990). On the other hand, *Malassezia furfur* can produce pityriacitrin, a UV-filtering compound believed to be protective (Machowinski et al. 2006). It is hypothesized that this fungus developed the UV-filter compound to reduce UV damage and compete for survival over other commensals (Machowinski et al. 2006). However, they did not find any adverse effect of pityriacitrin on commensals such as *S. aureus*, *S. epidermidis*, or *Candida albicans* (Machowinski et al. 2006).

### Odor Production

The metabolic activities of some skin microbes produce special odors. For example, human body odor is believed to result from bacterial growth and decomposition of secretions from specialized glands in the axillary region (Lam et al. 2018; Decréau et al. 2003; Natsch et al. 2003). Microbes are present in specific scent glands or tissue in mammals and modulate specific odors (Ezenwa et al. 2012). Skin microbes metabolize host sweat and produce volatile metabolites, enhancing the attractiveness of human sweat for the malaria mosquito (Brouwer 1960; Takken and Kline 1989). A recent study specified acetophenone, a volatile from the skin microbiota, promoted mosquito attractiveness in flavivirus-infected hosts (Zhang et al. 2022).

Skin commensal *Moraxella osloensis* (Li et al. 2021), a species highly tolerant to desiccation and UV irradiation, existed in various living environments, particularly in the laundry. This species has the potential to generate 4-methyl-3-hexenoic acid (4M3H), which is often described as a “wet-and-dirty-dustcloth-like malodor” or an “acidic or sweaty odor” (Kubota et al. 2012). In addition to bacteria, fungi are important sources of many volatile organic compounds (VOCs), including alcohols, aldehydes, esters, FAs, and terpenes (Belinato et al. 2019). In malignant fungating wounds (MFWs), metabolites such as dimethyl trisulfide (DMTS), four fatty acid volatiles (acetic acid, isobutyric acid, butyric acid, and isovaleric acid) and putrescine are linked with components of malignant fungating wound odor (Vardhan et al. 2019).

### Wound Healing

Wound healing is a complex but highly regulated process critical for skin barrier function (Han and Ceilley 2017). The presence and abundance of microbes in skin wounds depend on wound type (chronic/acute wound) (Johnson et al. 2018) and shifts over time (Loesche et al. 2017). Studies demonstrated that skin microbiota was also involved in wound healing in multifaceted ways. *S. epidermidis* promotes rapid KC progression via upregulation of TLR and downstream modulation of TNF-α in skin CD8^+^ T cells (Linehan et al. 2018; Naik et al. 2015). A study with a wound-induced hair follicle neogenesis (WIHN) mouse model revealed that skin microbiota promoted skin regeneration via IL-1β and KC-dependent IL-1R-MyD88 signaling (Wang et al. 2021). Metabolites from microbiota promote wound healing, e.g., lipoteichoic acid from *S. epidermidis* can decrease inflammation via TLR2 signaling (Lai et al. 2009). On the other hand, some potential pathogens do not promote cutaneous wound healing. For example, *S. aureus* (Kirker et al. 2009; den Reijer et al. 2016), *Acinetobacter. baumanni* and *A. junii* (de Breij et al. 2012) form biofilms on the SC and have a detrimental effect on human dermal fibroblast migration and ultimately result in cellular apoptosis (Kirker et al. 2012). Microbial stability was believed to be essential for skin health; however, temporal stability in the chronic wound is associated with poor healing as instability in the microbiome reflects effective control of wound bacteria, which prevents any community structure from stabilizing (Loesche et al. 2017).

## Conclusion

The present review centers on the current knowledge on skin microbiome from a perspective of skin as an ecosystem and tries to explore the fundamental driving force for the establishment and the balance of the highly personalized microbial feature. We believe that microenvironments that define the physical (e.g., pH, oxygen) and chemical (carbon sources and metabolites) conditions drive the microbiome composition. In turn, these microbes may reshape this environment via microbe–microbe or microbe–host interactions. Skin surface metabolome may be a critical approach to address causative correlations between the skin microbiome and skin phenome; therefore, future skin microbiome research should leverage those multi-omics to reveal these strong correlations and then validate them with the principle of Koch’s postulates. Furthermore, considering the higher complexity of the system due to the host genome and exposome, the longitudinal time-series study should be taken more into consideration for the control of these variables and for addressing the direction of those networks. Based on solid causative correlations, we can develop accurate interventions targeting specific skin microbe(s) and eventually reshape the skin conditions.

Of note, recent studies revealed that microbiota at strain level varies in the local microenvironment (Conwill et al. 2022), suggesting studies on higher resolution should be emphasized, which means deeper sequencing until strain level and more refined sampling sites up to single pore level. However, the greatest challenge for these designs is biomass, including metabolites and metagenomic biomass. This strongly relies on the technology development and iterative update of detection instruments to improve the sensitivity.

The significance of the human skin microbiome is increasingly appreciated. The approach from metagenomic sequencing (profiling) was gradually shifted to isolation/culturomics and function validation (mechanisms). However, some significant issues still exist, such as the lack of ideal *ex-vivo* skin models (e.g., reconstructed human epidermis (RHEs) and skin explants) that can reliably simulate the complexity of the host–microbe interactions (Harris-Tryon and Grice 2022; Larson et al. 2021). Some recent studies performed the function experiments with three-dimensional (3D) human skin equivalent. For example, a study using 3D skin tissue cultures revealed that a model microbiome or a mixed community of skin microbiome representatives led to pronounced changes in epidermal thickness, epidermal cell proliferation, and filaggrin production (Loomis et al. 2021). Another study investigated the interaction between the skin microbiota and environmental pollutant benzo[a]pyrene (B[a]P), with a microbially competent 3D skin model and demonstrated that commensal metabolism of xenobiotics can influence host toxicity (Lemoine et al. 2021). However, the limitations of these *ex-vivo* skin models are apparent, i.e., the lack of the histological/physiological/immunological complexity of RHEs, the paucity of inter-donor variability of skin explants, as well as short lifespan and the relatively high costs (Larson et al. 2021). Nevertheless, this is a matter of time to address these issues and push forward the skin microbiota targeted new intervention based on solid experimental evidence.

## Data Availability

Not applicable.
